# Drilling High Precision Holes in Ti6Al4V Using Rotary Ultrasonic Machining and Uncertainties Underlying Cutting Force, Tool Wear, and Production Inaccuracies

**DOI:** 10.3390/ma10091069

**Published:** 2017-09-12

**Authors:** M. A. K. Chowdhury, A. M. M. Sharif Ullah, Saqib Anwar

**Affiliations:** 1 Industrial Engineering Department, College of Engineering, King Saud University, P.O. Box 800, Riyadh 11421, Saudi Arabia; sanwar@ksu.edu.sa; 2 Faculty of Engineering, Kitami Institute of Technology, 165 Koen-cho, Kitami, Hokkaido 090-8507, Japan; ullah@mail.kitami-it.ac.jp

**Keywords:** rotary ultrasonic machining, drilling, Ti6Al4V, possibility distribution, uncertainty quantification

## Abstract

Ti6Al4V alloys are difficult-to-cut materials that have extensive applications in the automotive and aerospace industry. A great deal of effort has been made to develop and improve the machining operations of Ti6Al4V alloys. This paper presents an experimental study that systematically analyzes the effects of the machining conditions (ultrasonic power, feed rate, spindle speed, and tool diameter) on the performance parameters (cutting force, tool wear, overcut error, and cylindricity error), while drilling high precision holes on the workpiece made of Ti6Al4V alloys using rotary ultrasonic machining (RUM). Numerical results were obtained by conducting experiments following the design of an experiment procedure. The effects of the machining conditions on each performance parameter have been determined by constructing a set of possibility distributions (i.e., trapezoidal fuzzy numbers) from the experimental data. A possibility distribution is a probability-distribution-neural representation of uncertainty, and is effective in quantifying the uncertainty underlying physical quantities when there is a limited number of data points which is the case here. Lastly, the optimal machining conditions have been identified using these possibility distributions.

## 1. Introduction

Ti6Al4V is an important alloy material extensively utilized in many engineering industries such as spacecraft, aircraft, military and medical prosthesis. This is owing to the superior properties of Ti6Al6V. For instance, its high strength to weight ratio, high strength at elevated temperature, corrosion and oxidation resistance, good creep and fatigue strength, chemical inertness, fabricability and stability [1,2]. However, Ti6Al4V is regarded as a difficult-to-cut material due to work hardening, poor thermal conductivity, chemical reactivity with tool material at elevated temperature, saw-tooth chip formation, and low young modulus [2,3]. For machining of Ti6Al4V, rotary ultrasonic machining (RUM) exhibits high potential when compared to other machining methods, as demonstrated in previous studies [1,2,4,5,6]. Rotary ultrasonic machining consists of the material removal mechanisms of diamond grinding and ultrasonic machining. A rotating and ultrasonically vibrating diamond abrasives bonded tool is axially fed toward the work piece. Coolant is pumped through the tool in RUM to assist in keeping the tool cool and flushing the debris generated during machining.

Various studies have been reported using ultrasonic assisted RUM for drilling and grinding of various types of hard and brittle materials. Churi et al. [1] used RUM for drilling holes in Ti6Al4V alloy and studied the effects of spindle speed, feed rate and ultrasonic power on the cutting force, material removal rate and surface roughness. They reported that spindle speed, feed rate and ultrasonic power have significant effects on cutting force and surface roughness. Cong et al. [7] employed RUM for drilling holes in carbon fiber reinforced plastic composites and investigated the effect of cutting fluid and cold air on the cutting force, torque, surface roughness and tool wear. Zhang et al. utilized RUM for drilling holes in K9 glass and compared the results with diamond drilling [8]. The results showed that the rotary ultrasonic drilling produced significantly less fracturing of the workpiece surface and much lower forces when compared with diamond drilling. Li et al. [9] reported that a reduction of 50% in the cutting forces and an increase of 10% in material removal rate could be achieved during RUM of ceramic matrix composites, as compared to diamond drilling. In another study, Cong et al. [10] developed and validated a cutting force prediction model of RUM for carbon fiber reinforced plastics, assuming that brittle fracture is the dominant factor in material removal. The prediction trends by this model showed that cutting force decreases with the increase in ultrasonic vibration amplitude, tool rotation speed and abrasive size, and with the decrease in feed rate and abrasive concentration. Pujana et al. [5] studied ultrasonic assisted drilling of Ti6Al4V and reported higher force reductions and higher temperature increments with the increase in vibration amplitude. Choi et al. [11] applied RUM for grinding Ti6Al4V, FCD700 and S45C materials and reported that lower cutting temperature and tool wear are observed, when compared with conventional grinding. Tsai et al. [12] investigated the use of ultrasonic assisted end milling for the improvement of the machined surface quality of hard Stavax mold steel. They found that an optimum amplitude value of the ultrasonic vibrations is to be used for the best surface finish, and that increasing the amplitude does not improve surface quality. Wang et al. [13] developed a novel edge chipping mechanism for the drilling of holes considering machining-induced cracks, and the model was verified by experiments on quartz glass. The study revealed that both the magnitude of the driving force and the size of the machining-induced crack determine the initiation of edge chipping. Liu et al. proposed a mechanistic cutting force model for RUM of brittle materials [14]. The input variables of this model are core drill variables, properties of work-piece material, ultrasonic vibration variables and machining process variables. The developed model was verified by the experiments on alumina. Noma et al. [15] demonstrated that the drilled hole quality, in terms of exit chip size, can be significantly improved by the application of the compressive stresses at the bottom surface of the rotary ultrasonic machined chemically strengthened glass. In another study, Nambu et al. demonstrated that the ratio of the hole depth to drill diameter during micro hole drilling can be significantly increased by the application of ultrasonic vibrations to the drill tool [16]. It is observed that in reported studies, the performance of the RUM machining process has been evaluated in terms of cutting force, material removal rate, exit chip size, temperature generation and tool wear. The results of these studies have proven the benefits of RUM in terms of lower cutting forces and temperatures, higher material removal rates and quality of the drilled holes when compared with the available alternative machining processes.

Tool wear is a critical factor in RUM, which influences geometrical accuracy and the material removal rate of RUM [17]. Dam et al. [18] stated that the hardness and toughness properties of materials affect tool wear in RUM. They reported that higher fracture toughness and hardness of ceramic work material increases tool wear. Kumar et al. [19] performed an experimental study to find out the tool wear rate in ultrasonic machining of pure titanium. They identified that power rating and tool material are the most influential parameters for the variation in the tool wear rate. Jadoun et al. [20] used tools of tungsten carbide, high carbon steel and high speed steel to investigate the tool wear rate in ultrasonic drilling of ceramics. Less tool wear is found in tools made of tungsten carbide, compared to high speed steel and high carbon steel. 

Kumar [21] reported that geometrical inaccuracies in ultrasonic machining can be categorized as dimensional inaccuracy (overcut) and form inaccuracy (conicity). He also added that a decrease in abrasive grain size leads to improved accuracy of drilled holes in ultrasonic machining. Adithan and Venkatesh [22] showed that higher static load and machining time increases the overcut error and conicity error for glass material in ultrasonic machining. They also demonstrated that overcut error is greatest at entry, and this error increases with increases in diameter-length ratio in ultrasonic drilling. They added that grain structure and brittle fracture properties of work material also influence the overcut and conicity. Komaraiah et al. [23] stated that increases in the ratio of hardness to Young’s modulus of elasticity of work material results in increases of out-of-roundness of the drilled holes in ultrasonic machining. They also reported that RUM shows better performance for out-of-roundness results compared to ultrasonic machining. Ding et al. [24] performed an experimental study on drilling holes in C/SiC composites using RUM, and reported that RUM is a useful machining process for reduction of tearing defects. Feng et al. [25] investigated the tearing defect formation during drilling holes in C/SiC composites using RUM. They found that the reduction of thrust force (the drilling force along the axial direction) results in decreased tearing defects at the hole exit by more than 60%. They also reported that the tearing defect can be further decreased by decreasing the feed rate or by increasing the spindle speed and ultrasonic amplitude.

Before now, no work has been reported to study the effect of RUM machining parameters on the performance parameters of cutting force, tool wear, and production inaccuracies—simultaneously for drilling holes in Ti6Al4V—using a systematically designed experimental approach. In drilling holes, the important production inaccuracies are overcut error (cutting inaccuracy) and cylindricity error (form inaccuracy). The current study attempts to experimentally analyze the RUM parameters for drilling high precision holes in Ti6Al4V. The main aim of the current study is to systematically present the effects of the machining conditions (ultrasonic power, feed rate, spindle speed and tool diameter) on the performance parameters (cutting force, tool wear, overcut error and cylindricity error) by building a set of possibility distributions (i.e., trapezoidal fuzzy numbers). Later, the optimal cutting conditions are identified using the possibility distributions.

## 2. Experimentation

**Figure 1:**
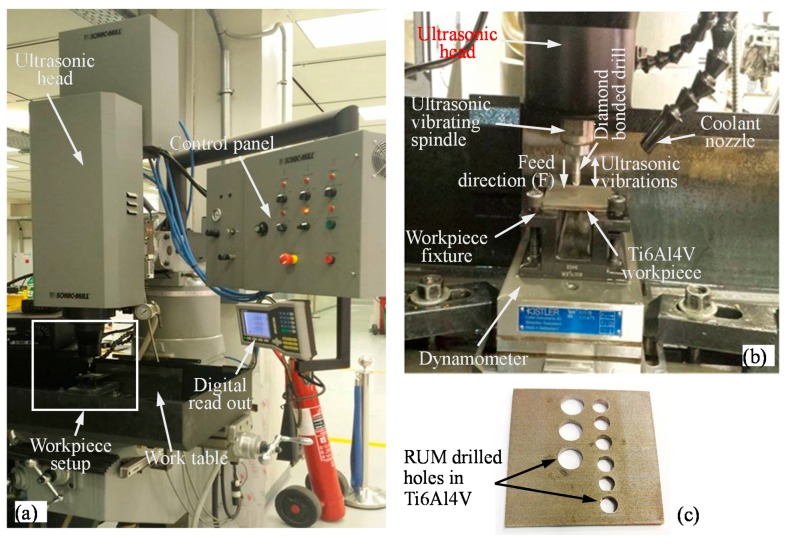
(**a**) Rotary ultrasonic machine used for experiments; (**b**) Rotary ultrasonic machining setup; (**c**) A Ti6Al4V work piece with drilled holes.

Machining experiments are performed on Sonic-mill series 10 rotary ultrasonic machines (SONIC-MILL, Albuquerque, NM, USA), with a maximum spindle rotation speed of 8000 rpm, maximum vibration frequency of 20 kHz, and maximum ultrasonic power of 1000 Watts. The rotary ultrasonic machining setup which was used for drilling holes in Ti6Al4V work pieces is shown in Figure 1. The three main systems of rotary ultrasonic machining setup are an ultrasonic spindle system, a coolant set-up and a data acquisition system. The main components of the ultrasonic spindle system are an ultrasonic spindle, a power supply, a motor and a speed controller. The electric supply is converted into a high-frequency (20 kHz) electrical signal by the power supply. The piezoelectric actuator transforms high-frequency electrical signals to mechanical vibrations along the tool feed direction. The ultrasonic vibrations are then amplified and transmitted to the spindle. The amplitude of the tool vibrations can be changed by regulating the ultrasonic power supply output. The spindle motor speed controller can be adjusted to vary the rotational speed of the spindle. These motions of the spindle provide the drill tool rotational motion and ultrasonic vibration. The drill is then fed into the workpiece with a constant pressure or constant feed rate. The cooling system supplies the coolant at the interface of the diamond tool and work piece to reduce the cutting temperature and to flush out the debris. In this setup, water miscible Fuchs Ecocool S-HL oil (Fuchs, Dissen, Germany) (with a concentration of 10%) was used as coolant.

In this research, Ti6Al4V supplied by Magellan (Norwalk, CT, USA) is used for drilling holes. The chemical composition and mechanical properties of Ti6Al4V are given in Table 1 and Table 2, respectively. The dimensions of the work piece used in experiments are 50 mm × 50 mm × 3 mm. All the produced holes in work pieces are through drilled.

**Figure 2:**
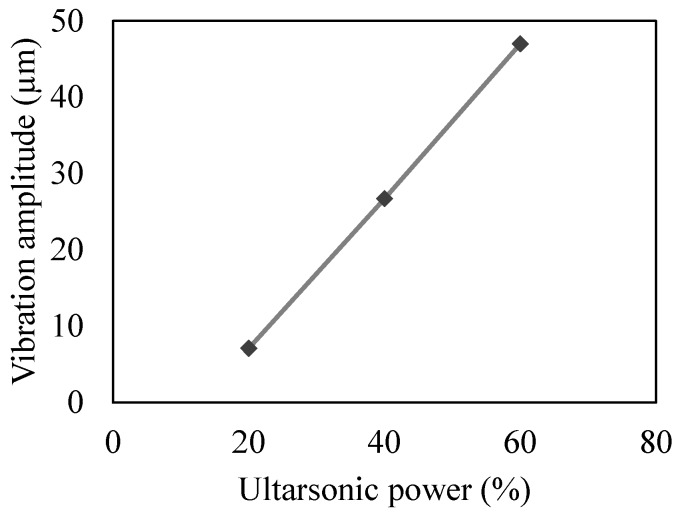
Relationship between ultrasonic power (%) and vibration amplitude (μm).

The input parameters that are varied during the experiments include ultrasonic power supply, feed rate, spindle speed and tool diameter. Due to the limitation of the Sonic-mill machine used during the experiments, the frequency of the ultrasonic vibrations was kept constant at 20 KHz. The ultrasonic power was varied to change the amplitude of the ultrasonic vibrations [26,27,28]. The relationship between the ultrasonic power and the vibration amplitude is shown in Figure 2. A single input parameter is changed at a time while others are kept constant. Each trial is repeated two times and an average output response is calculated. The input parameters and their respective values are shown in Table 3.

**Figure 3:**
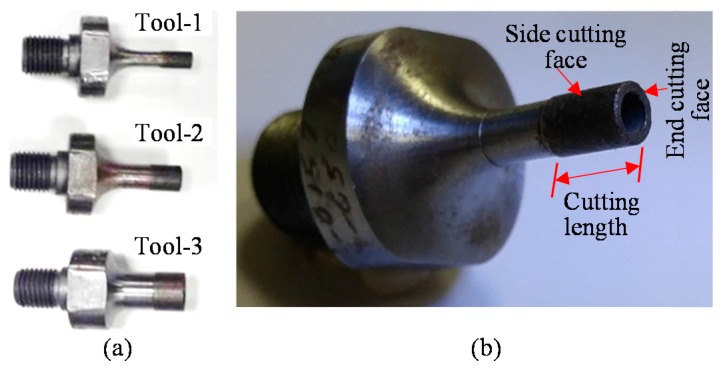
(**a**) Three different diameters of metal bonded diamond drills used in experiments; (**b**) Zoomed-in view of a metal bonded drill.

**Figure 4:**
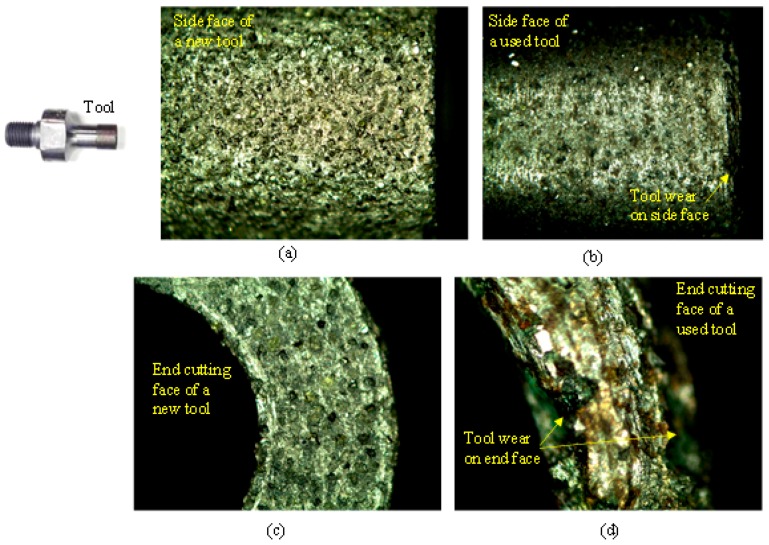
(**a**) Zoomed-in view of the side cutting face of a typical new drill; (**b**) Zoomed-in view of the side cutting face of a drill after machining; (**c**) Zoomed-in view of the end cutting face of a typical new drill; (**d**) Zoomed-in view of the end cutting face of a drill after machining.

The cutting tools used are hollow metal bonded diamond core drills (Sonic Mill, Albuquerque, NM, USA). Three different diameter tools are used in the experiments, as shown in Figure 3a,b and enlarged side cutting faces and end cutting faces of new and used tools are presented in Figure 4a–d. The cutting length for all the tools is 12.6 mm. The mesh size of the diamond abrasives on all the tools is 80–100 μm.

Kistler dynamometer type 9257B with charge amplifier type 5070A and data acquisition type 5697A1 (Kistler Corp, Winterthur, Switzerland) were used to measure the cutting force (*F_C_*) along the feed direction during drilling. Voltage signals generated from dynamometer during RUM of samples are amplified by the charge amplifier. Later, the data acquisition system converts the amplified signals into numerical signals. To measure the drilled holes’ quality, a coordinate measuring machine (CMM, Zeiss Accura, Oberkochen, Germany) was used to measure the holes’ cylindricity error and overcut error. For both cylindricity error and overcut error, twenty-seven points were measured on the cylindrical surface of the holes at three depths; nine points measured at each depth level across the circular profile. The tool wear is defined as the weight loss of the cutting tool during machining of each hole. Specifically, it is the difference between tool weights before and after drilling a hole. After drilling of each hole, the cutting tool was detached to clean the residual by acetone [26,27]. A high-accuracy weight balance (Model PW124 Analytical Balance, Adam Equipment, Oxford, UK) was used to measure the weight of the tool.

The design of experiments is considered to be a very useful method for deducing accurate and meaningful inferences from the experimental data. In the current study, a L36 design of experiments is implemented to capture the effect of the RUM variables on the four output responses: cutting force (*F_C_*), tool wear (*T_W_*), overcut error (*O_E_*) and cylindrical error (*C_E_*). The variable input parameters and their respective levels are shown in Table 3. The levels of each input parameter are used, leading to a total of 36 experiments, as shown in Table 4.

## 3. Uncertainty Analysis

This section describes an uncertainty analysis that helps build the relationship between the machining conditions and machining performances. The goal is to predict the machining performances beforehand. Here, the machining conditions mean values of parameters such as Ultrasonic Power (*P*), Feed Rate (*F*), Spindle Speed (*S*), and Tool Diameter (*D*), as listed in Table 3. The machining performances mean the values of parameters such as Cutting Force (*F_C_*), Tool Wear (*T_W_*), Over Cut Error (*O_E_*), and Cylindricity Error (*C_E_*), as listed in Table 4. The experimental results shown in Table 4 become the input information for the uncertainty analysis that exhibits the relationships among machining conditions (*P*, *F*, *S*, and *D*) and machining performances (*F_C_*, *T_W_*, *O_E_*, and *C_E_*).

It is worth mentioning that the relationships can be expressed in terms of some “if…then…” rules [29,30]. Alternatively, one can use possibility distributions or fuzzy numbers (e.g., triangular fuzzy numbers and trapezoidal fuzzy numbers) [31,32,33,34] to represent the uncertainty associated with each combination of machining condition and performance parameter. Possibility distribution based analysis has been shown to be effective in quantifying the uncertainty associated with materials’ properties [35,36,37], surface roughness [38,39], CO2 emissions and alike [40]. A possibility distribution is a probability-distribution-neutral representation of uncertainty associated with a quantity [31,32,33,34]. It is particularly suitable for quantifying the uncertainty when there is a lack of data [37]. As such, it is appropriate for this particular case. For every given state of machining condition, there are only 12 sets of data points for each performance variable. The methodology to induce a possibility distribution from a given set of numerical data points described in [33] is used in this study to quantify the uncertainty in the performance variable for each state of machining conditions (see Appendix A).

**Figure 5:**
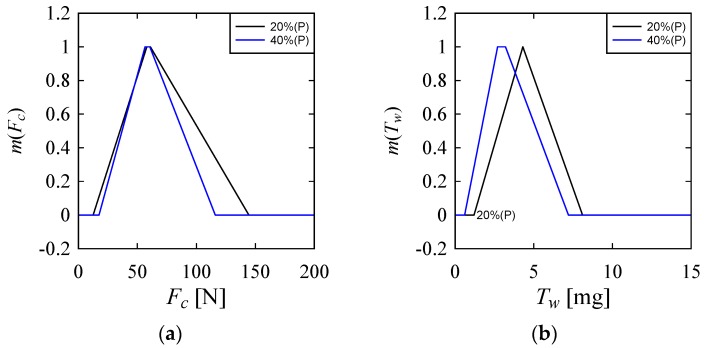

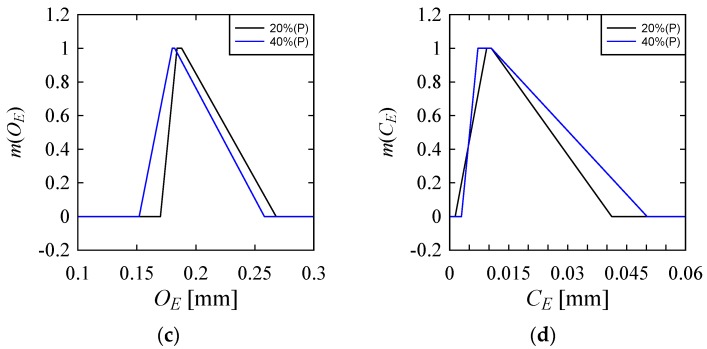
Effect of power on the performance parameters (**a**) On Cutting Force (*F_C_*); (**b**) On Tool Wear (*T_W_*); (**c**) On Over Cut Error (*O_E_*); (**d**) On Cylindricity Error (*C_E_*).

**Figure 6:**
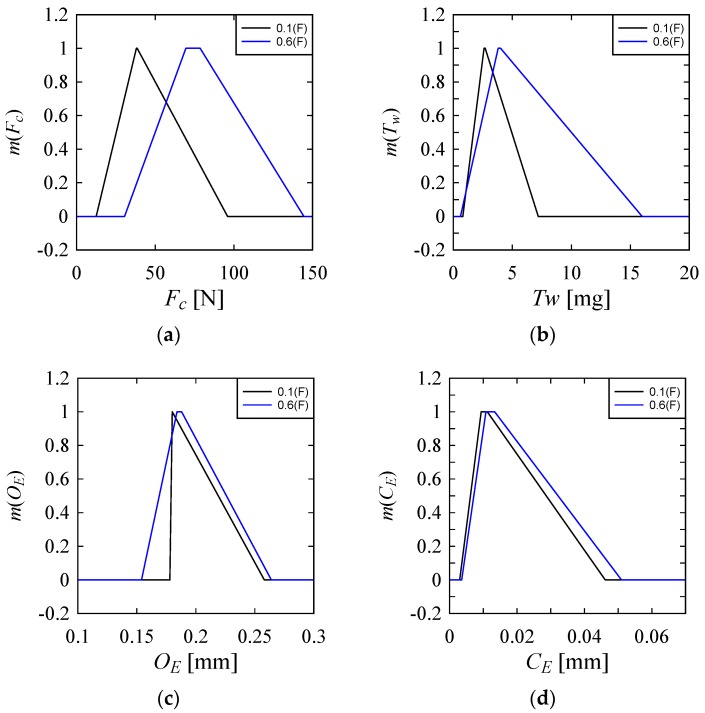
Effect of feed on the performance parameters (**a**) On Cutting Force (*F_C_*); (**b**) On Tool Wear (*T_W_*); (**c**) On Over Cut Error (*O_E_*); (**d**) On Cylindricity Error (*C_E_*).

**Figure 7:**
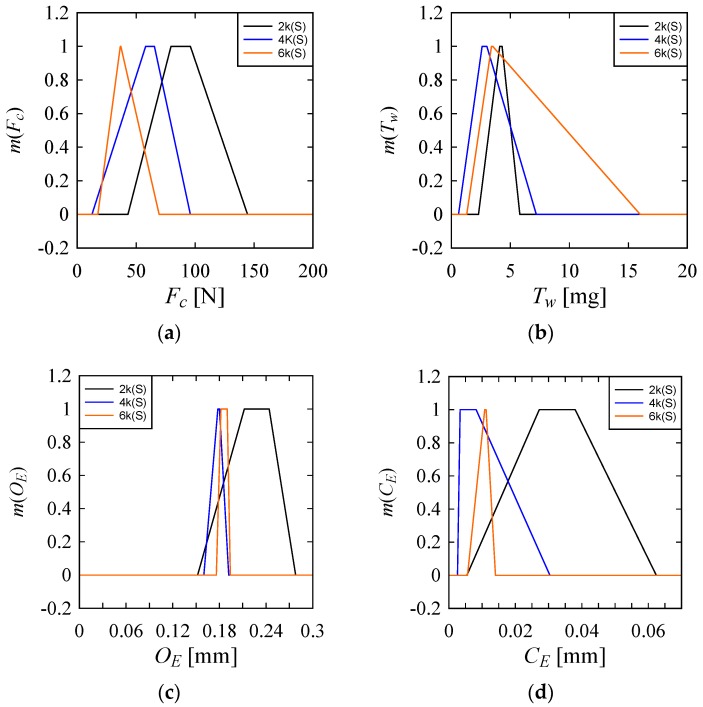
Effect of spindle speed on the performance parameters (**a**) On Cutting Force (*F_C_*); (**b**) On Tool Wear (*T_W_*); (**c**) On Over Cut Error (*O_E_*); (**d**) On Cylindricity Error (*C_E_*).

**Figure 8:**
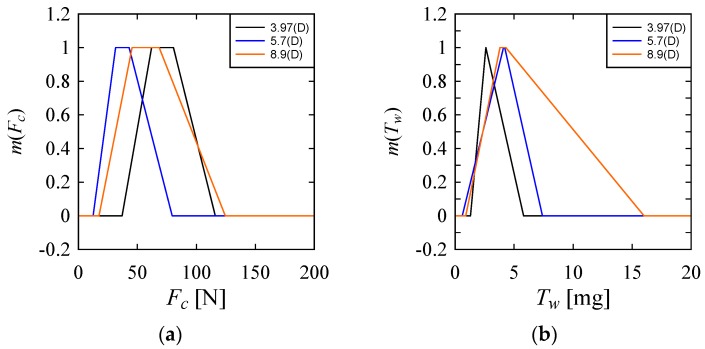

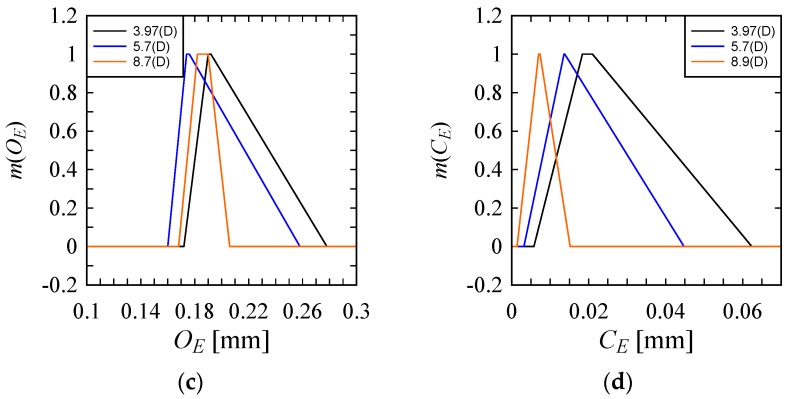
Effect of tool diameter on the performance parameters. (**a**) On Cutting Force (*F_C_*); (**b**) On Tool Wear (*T_W_*); (**c**) On Over Cut Error (*O_E_*); (**d**) On Cylindricity Error (*C_E_*).

Figure 5a–d shows the results. Figure 5 shows the effect of *P* on *F_C_*, *T_W_*, *O_E_*, and *C_E_*; Figure 6 shows the effect of *F* on *F_C_*, *T_W_*, *O_E_*, and *C_E_*; Figure 7 shows the effect of *S* on *F_C_*, *T_W_*, *O_E_*, and *C_E_*; Figure 8 shows the effect of *D* on *F_C_*, *T_W_*, *O_E_*, and *C_E_*. The caption of vertical axis in all plots in Figure 5a–d is the membership values of the respective possibility distributions, which is always a number in the interval [0, 1]. See [31,32,33] for more details about the properties of possibility distributions. As seen in Figure 5a–d, compared to *P* = 20%, *P* = 40% exhibits a somewhat better control over *Fc*, *T_W_* and *O_E_*. On the other hand, compared to *P* = 40%, *P* = 20% exhibits a somewhat better control over *C_E_*. Having said that, it is also true that neither an increase or decrease of *P* ensures the performance to a stipulated range. A very similar trend is seen for the possibility distributions of *F*, as shown in Figure 6. Having said that, it is also true that *F* = 0.1 mm/min helps keep the *F_C_* low, as is evident from the possibility distribution of *F_C_* in Figure 6.

However, (as shown in Figure 7) *S* = 6000 rpm is effective for having low *F_C_* and *C_E_*; *S* = 4000 rpm or 6000 rpm is effective for having low *O_E_*. On the other hand, to keep *T_W_* to a narrow range, *S* = 2000 rpm is the best but *S* = 4000 also results in low *T_W_*.

Referring to Figure 8, *D* = 5.7 mm ensures low *F_c_*, compared to *D* = 3.97 mm and 8.7 mm. As far as a low *T_w_* is concerned, *D* = 3.97 mm is recommended. As far as a low *O_E_* or *C_E_* is concerned, *D* = 8.9 mm is the best.

The abovementioned uncertainty analysis results in an optimization procedure described in Table 5. The combinations of machining conditions shown in Table 5 can be used to perform machining. These combinations will help minimize all the performance parameters.

## 4. Concluding Remarks

(1) This study investigated the effect of the machining conditions on the performance parameters for drilling high precision holes in Ti6Al4V using rotary ultrasonic machining. It was found that increases or decreases of power did not ensure a specific performance. Low feed is good for reducing cutting force and it also ensures low tool wear. High spindle speed is good for having low cutting force, and increase in spindle speed reduces cutting force. Low or moderate spindle speed is good for reducing tool wear. However, high spindle speed ensures low overcut error and low cylindrical error. It was also observed that smaller tool diameter ensures low tool wear, and bigger tool diameter ensures low overcut error and cylindrical error.
(2) This study also depicts the uncertainty associated with the performance parameters for drilling in Ti6Al4V by rotary ultrasonic machining using possibility distributions. Finally, the optimal machining conditions are identified using possibility distributions, and it can be stated that different sets of machining conditions are required to minimize different performance parameters.

## Figures and Tables

**Table 1:** Chemical composition of the Ti6Al4V.

Constituent	Ti	Al	V	Fe	Cu	Mu	Mo
Composition %	Balance	6.35	4.01	0.167	<0.005	<0.01	<0.005

**Table 2:** Mechanical properties for Ti6Al4V.

Property	Value
Thermal conductivity (W·m^−1^·K^−1^)	21
Tensile strength (GPa)	950
Rockwell hardness (HRC)	40
Density (Kg·m^−3^)	4510
Melting point (K)	1941 ± 285
Coefficient of thermal expansion (K^−1^)	8.64 × 10^−6^

**Table 3:** Machining parameters and their selected values.

Input Parameter	Abbreviation	Level 1	Level 2	Level 3
Ultrasonic power	*P*	20%		40%
Feed rate (mm/min)	*F*	0.1		0.6
Spindle speed (rev/min)	*S*	2000	4000	6000
Tool diameter (mm)	*D*	3.97	5.9	8.9

**Table 4:** Design of experiments and corresponding results.

Exp. No	Ultra-Sonic Power (*P*) %	Feed Rate (*F*) mm/min	Spindle Speed (*S*) rev/min	Tool Diameter (*D*) mm	Cutting Force (*F_C_*) N	Tool Wear (*T_W_*) mg	Over Cut Error (*O_E_*) mm	Cylindricity Error (*C_E_*) mm
1	20	0.1	2000	3.97	97.32	2.8	0.2787	0.0463
2	20	0.1	2000	5.9	67.58	0.9	0.2488	0.0251
3	20	0.1	2000	8.9	30.2	4.5	0.1824	0.0152
4	20	0.1	4000	3.97	96.41	6.4	0.1762	0.0093
5	20	0.1	4000	5.9	12.8	4.4	0.1793	0.0037
6	20	0.1	4000	8.9	13.85	7.2	0.1549	0.0086
7	20	0.1	6000	3.97	62.1	2.6	0.1922	0.0096
8	20	0.1	6000	5.9	13.75	1.5	0.1745	0.0062
9	20	0.1	6000	8.9	21.94	1.9	0.1803	0.0053
10	20	0.6	2000	3.97	158.62	2.6	0.2645	0.0384
11	20	0.6	2000	5.9	144.76	7.4	0.2697	0.0272
12	20	0.6	2000	8.9	124.75	4.9	0.2122	0.0012
13	20	0.6	4000	3.97	58.3	3.2	0.2152	0.0412
14	20	0.6	4000	5.9	30.54	8.1	0.1931	0.0304
15	20	0.6	4000	8.9	69.7	3.7	0.1852	0.002
16	20	0.6	6000	3.97	36.83	5.1	0.1925	0.0186
17	20	0.6	6000	5.9	31.63	5.4	0.1771	0.0073
18	20	0.6	6000	8.9	99.84	16	0.2075	0.011
19	40	0.1	2000	3.97	56.53	2.8	0.2854	0.0658
20	40	0.1	2000	5.9	44.21	4.7	0.2593	0.0448
21	40	0.1	2000	8.9	45.89	5.1	0.1985	0.0152
22	40	0.1	4000	3.97	88.4	2.7	0.1885	0.0074
23	40	0.1	4000	5.9	17.2	1.6	0.1798	0.0035
24	40	0.1	4000	8.9	39.72	1.1	0.1737	0.003
25	40	0.1	6000	3.97	38.11	1.6	0.1947	0.0143
26	40	0.1	6000	5.9	23.86	7.2	0.1792	0.014
27	40	0.1	6000	8.9	18.8	3.5	0.1809	0.0116
28	40	0.6	2000	3.97	116	5.8	0.1196	0.0624
29	40	0.6	2000	5.9	79.92	4.2	0.1562	0.0511
30	40	0.6	2000	8.9	104.36	3.9	0.1964	0.0059
31	40	0.6	4000	3.97	81.66	0.7	0.1922	0.0032
32	40	0.6	4000	5.9	66.88	0.9	0.1654	0.0041
33	40	0.6	4000	8.9	141.5	1	0.1858	0.0072
34	40	0.6	6000	3.97	61.71	2.4	0.1838	0.0214
35	40	0.6	6000	5.9	49.9	3.7	0.1731	0.0139
36	40	0.6	6000	8.9	69.55	24.5	0.1941	0.0078

**Table 5:** Optimal machining conditions.

Minimize	*P* (%)	*F* (mm/min)	*S* (rev/min)	*D* (mm)
*F_C_* (N)	40	0.1	6000	5.7
*T_w_* (mg)	40	0.1	4000	3.97
*O_E_* (mm)	40	0.1	6000	8.9
*C_E_* (mm)	20	0.1	2000	8.9

**Figure A1:**
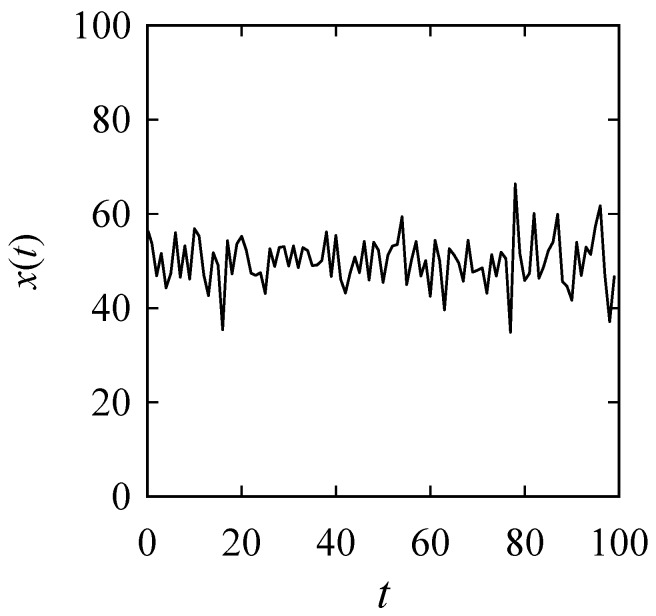
A given set of numerical data.

**Figure A2:**
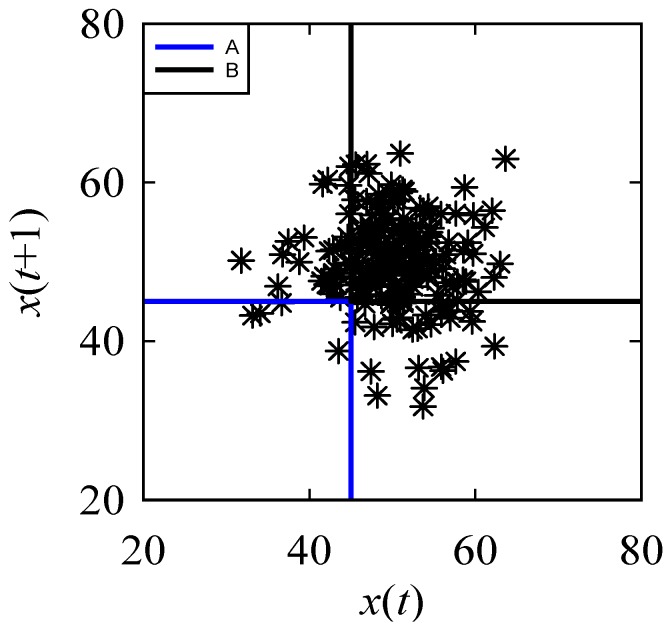
Relative position of *A* and *B* in the point-cloud (*x*(*t*), *x*(*t* + 1)).

**Figure A3:**
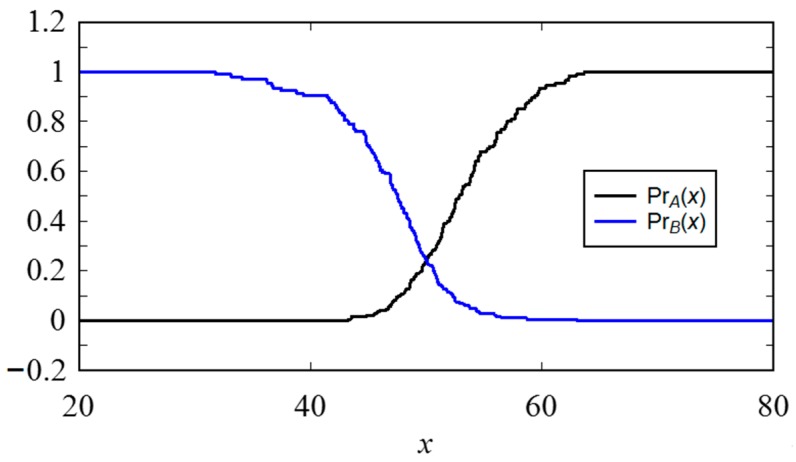
The typical nature of Pr*_A_*(*x*) and Pr*_B_*(*x*) for unimodal quantity.

**Figure A4:**
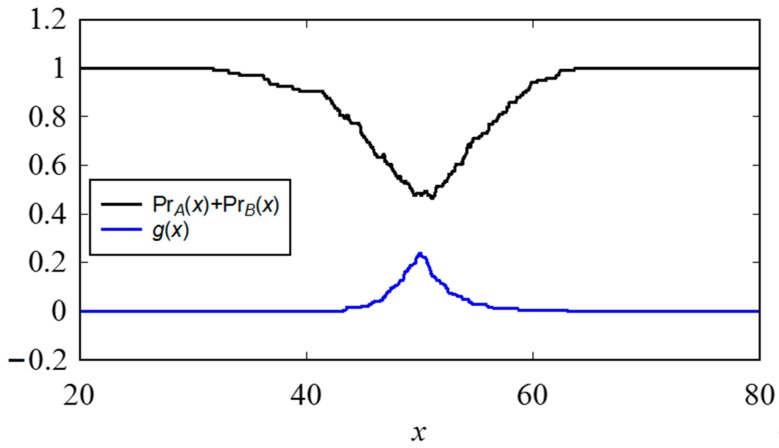
Nature of Pr*_A_*(*x*) + Pr*_B_*(*x*) and min(Pr*_A_*(*x*), Pr*_B_*(*x*)) for unimodal data.

**Figure A5:**
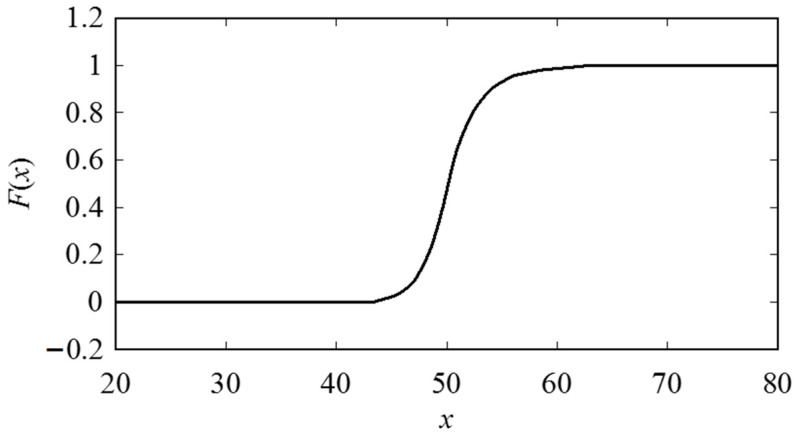
Nature of cumulative probability distribution of a point-cloud.

**Figure A6:**
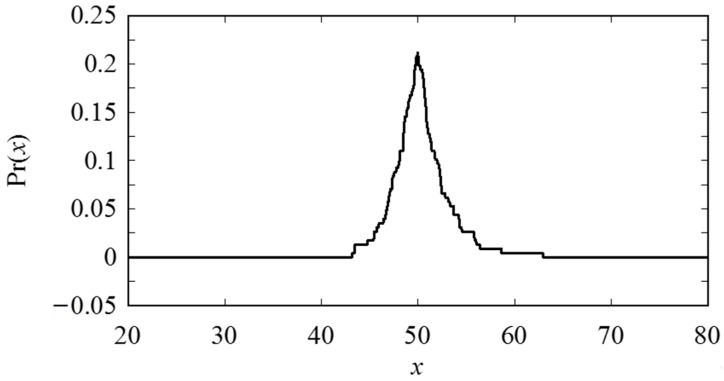
The nature of probability distribution of a unimodal point-cloud.

**Figure A7:**
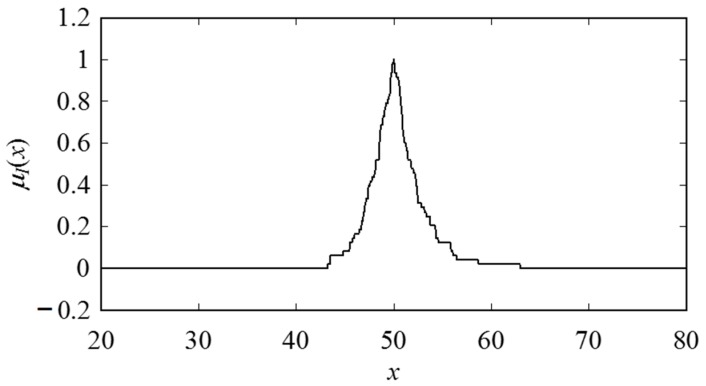
The nature of possibility distribution of a unimodal point-cloud.

## Appendix A. Inducing Possibility Distributions (Fuzzy Numbers) from Numerical Data

This appendix describes the mathematical procedures applied for inducing the possibility distributions from the numerical data reported in this paper, as follows.

Let *x*(*t*) ∈ ℜ, *t* = 0, …, *n* − 1 be *n* data points, as shown in Figure A1.

Let (*x*(*t*), *x*(*t* + 1)), *t* = 0, …, *n* − 1, be a point-cloud in the universe of discourse *X* = [*x*_min_, *x*_max_] so that *x*_min_ < min(*x*(*t*)|∀*t* ∈ {0, …, *n*}) and *x*_max_ > max(*x*(*t*)|∀*t* ∈ {0, …, *n*}). Let *A* and *B* two square boundaries so that the vectors of the vertices of *A* and *B* (in the anti-clockwise direction) are ((*x*_min_, *x*_min_), (*x*, *x*_min_), (*x*, *x*), (*x*_min_, *x*)) and ((*x*_max_, *x*_max_), (*x*, *x*_max_), (*x*, *x*), (*x*_max_, *x*)), respectively, ∀*x* ∈ *X*. As such, (*x*, *x*) is their common vertex of A and B. For example, consider the arbitrary point-cloud shown in Figure A2. As from Figure A2, the universe of discourse *X* = [20, 80]. Notice the relative positions of boxes denoted by *A* and *B* in Figure 2. The boxes are connected at their common vertices.

Let Pr*_A_*(*x*) and Pr*_B_*(*x*) be two subjective probabilities, wherein Pr*_A_*(*x*) and Pr*_B_*(*x*) represent the degrees of chances that the points in the point-cloud are, in *A* and *B*, respectively. As such, these functions are defined by the following mappings:

(A1) $$\begin{array}{l} X\to \left[0,1\right]\\ x\mapsto {\mathrm{Pr}}_{A}\left(x\right)=\frac{\sum_{i=0}^{n-1}\Theta \left(t\right)}{n-1}\\ \Theta \left(t\right)=\left\{ \begin{array}{l} 1\;\left(\left(x\left(t\right)\leq x\right)\wedge \left(x\left(t+1\right)\leq x\right)\right)\\ 0\;otherwise \end{array}\right. \end{array}$$

(A2) $$\begin{array}{l} X\to \left[0,1\right]\\ x\mapsto {\mathrm{Pr}}_{B}\left(x\right)=\frac{\sum_{i=0}^{n-1}\Omega \left(t\right)}{n-1}\\ \Omega \left(t\right)=\left\{ \begin{array}{l} 1\;\left(\left(x\left(t\right)\geq x\right)\wedge \left(x\left(t+1\right)\geq x\right)\right)\\ 0\;otherwise \end{array}\right. \end{array}$$

The typical nature of the functions defined in Equations (A1) and (A2) are illustrated in Figure 3, using the information of the point-cloud shown in Figure A2. Note that Pr*_A_*(*x*) increases with the increase in *x* and the opposite is true for Pr*_B_*(*x*). It is worth mentioning that Pr*_A_*(*x*) + Pr*_B_*(*x*) ≤ 1 for the point-cloud, though for some cases Pr*_A_*(*x*) + Pr*_B_*(*x*) = 1 (see Figure 4). This means that Pr*_A_*(*x*) + Pr*_B_*(*x*) does not serve the role of “cumulative probability distribution.” A cumulative probability distribution can however be formulated by using the information of Pr*_A_*(*x*) and Pr*_B_*(*x*), as shown in Figure A3:

Consider a mapping that maps *x* into the minimum of Pr*_A_*(*x*) and Pr*_B_*(*x*), as follows:

(A3) $$\begin{array}{l} X\to \left[0,a\right]\\ x\mapsto g\left(x\right)=\min\left({\mathrm{Pr}}_{A}\left(x\right),{\mathrm{Pr}}_{B}\left(x\right)\right) \end{array}$$

In Equation (A3), *a* = 1, if the point-cloud is a point; otherwise, *a* < 1. Figure A4 shows the nature of *g*(*x*) for Pr*_A_*(*x*) and Pr*_B_*(*x*). The area under g(*x*) is given by:

(A4) $$Q=\int_{X}g\left(x\right)dx$$

There is no guarantee that *Q* = 1. Otherwise, *g*(*x*) could have been considered a probability distribution of the underlying point-cloud. However, a function *F*(*x*) can defined, as follows:

(A5) $$\begin{array}{l} \left[0,a\right]\to \left[0,1\right]\\ x\mapsto F\left(x\right)=\frac{\int_{x_{\min}}^{x}g\left(x\right)dx}{Q} \end{array}$$

*F*(*x*) can be considered a cumulative probability distribution because max(*F*(*x*)) = 1, *F*(*x*) ≥ *F*(*z*) for *x ≥ z*, *F*(*x*) ∈ [0, 1], ∀*x*, *z* ∈ *X*. Figure A5 shows the nature of *F*(*x*) derived from *g*(*x*) shown in Figure A4. The cumulative probability distribution defined in Equation (A5) produces a probability distribution Pr(*x*). Thus, the following formulation holds:

(A6) $$\mathrm{Pr}\left(x\right)=\frac{dF\left(x\right)}{dx}$$

Figure A6 shows the probability distribution Pr(*x*) underlying *F*(*x*) shown in Figure A5. It is needless to say that the area under the probability distribution Pr(*x*) is a unit and Pr(*x*) remains in the bound of [0, 1].

From the induced probability distribution Pr(*x*), a possibility distribution given by the membership function *μ_I_*(*x*)) can be defined based on the heuristic rule of probability-possibility transformation—that the degree of possibility is greater than or equal to the degree of probability. The easiest formulation is to normalize Pr(*x*) by its maximum value, max(Pr(*x*)|∀*x* ∈ *X*). Therefore,

(A7) $$\begin{array}{l} \left[0,1\right]\to \left[0,1\right]\\ \mathrm{Pr}\left(x\right)\mapsto \mu_{I}\left(x\right)=\frac{\mathrm{Pr}\left(x\right)}{\max\left(\mathrm{Pr}\left(x\right)|\forall x\in X\right)} \end{array}$$

Figure A7 shows the possibility distribution *μ_I_*(*x*) derived from the probability distribution Pr(*x*) shown in Figure 6. The shape of the induced probability distribution and the shape of the induced possibility distribution are identical, as evident from Figure A6 and Figure A7. Other formulations can be used instead of the formation (A7) as suggested by others.

However, it is observed that when the point-cloud resembles the point-cloud of a bimodal quantity, the induced possibility distribution resembles a trapezoidal fuzzy number. In addition, when the point-cloud is a point, the induced possibility distribution becomes a fuzzy singleton. Moreover, when the point-cloud resembles the point-cloud of a unimodal data, the induced probability/possibility distribution resembles a triangular fuzzy number. To define the membership function of an induced fuzzy number in the form of a triangular fuzzy number, the following formulation can be sued.

Let *u*, *v*, and *w* be three points in the ascending order in the universe of discourse *X*, *u* ≤ *v* ≤ *w* ∈ *X*. Let the interval [*u*, *w*] be the *support* of a triangular fuzzy number and the point *v* be the *core*. The following procedure can be used to determine the values of *u*, *v*, and *w* from the induced fuzzy number *μ_I_*(*x*) (Equation (A7)):

(A8) $$\begin{array}{l} u\leq v\leq w\in X\\ u=x\;\left(\mu_{I}\left(x\right)=0\wedge \mu_{I}\left(x+dx\right)>0\right)\\ v=x\;\left(\mu_{I}\left(x-dx\right)<1\wedge \mu_{I}\left(x\right)=1\right)\\ w=x\;\left(\mu_{I}\left(x-dx\right)>0\wedge \mu_{I}\left(x\right)=0\right) \end{array}$$

As defined in (A8), *u* is the point after which the membership value *μ_I_*(*x*) is greater than zero, *v* is the point corresponding to the maximum membership value max(*μ_I_*(*x*)), and *w* is the point from/beyond which the membership value *μ_I_*(*x*) again becomes/remains zero. Thus, the membership function *μ_T_*(*x*) of the induced triangular fuzzy number is as follows:

(A9) $$\begin{array}{l} X\to \left[0,1\right]\\ x\mapsto \mu_{T}\left(x\right)=\max\left(0,\min\left(\frac{x-u}{v-u},\frac{w-x}{w-v}\right)\right) \end{array}$$

It is needless to say that this formation is valid only for the point-cloud exhibiting the nature of an unimodal quantity.
